# Degradation and Toxicity Analysis of a Reactive Textile Diazo Dye-Direct Red 81 by Newly Isolated *Bacillus* sp. DMS2

**DOI:** 10.3389/fmicb.2020.576680

**Published:** 2020-09-24

**Authors:** Shivani Amin, Rajesh Prasad Rastogi, Mukesh Ghanshyam Chaubey, Kunal Jain, Jyoti Divecha, Chirayu Desai, Datta Madamwar

**Affiliations:** ^1^Post-Graduate Department of Biosciences, UGC-Centre of Advanced Study, Sardar Patel University, Satellite Campus, Bakrol, India; ^2^Department of Biotechnology, Shree A. N. Patel PG Institute of Science and Research, Sardar Patel University, Anand, India; ^3^Department of Statistics, Sardar Patel University, Vallabh Vidyanagar, India; ^4^P.D. Patel Institute of Applied Sciences, Charotar University of Science and Technology (CHARUSAT), Changa, India

**Keywords:** Direct Red 81, toxicity, *Caenorhabditis elegans*, *Lemna minor*, biodegradation, response surface methodology

## Abstract

An efficient diazo dye degrading bacterial strain, *Bacillus* sp. DMS2 was isolated from a long-term textile dye polluted environment. The strain was assessed for its innate ability to completely degrade and detoxify Direct Red 81 (DR81) textile dye under microaerophilic conditions. The degradation ability of strain showed significant results on optimizing the nutritional and environmental parameters. Based on statistical models, maximum efficiency of decolorization achieved within 24 h for 100 mg/l of dye supplemented with glucose (0.02%), MgSO_4_ (0.002%) and urea (0.5%) at 30°C and pH (7.0). Moreover, a significant catabolic induction of a laccase and azoreductase suggested its vital role in degrading DR81 into three distinct metabolites (intermediates) as by-products. Further, toxicity analysis of intermediates were performed using seeds of common edible plants, aquatic plant (phytotoxicity) and the nematode model (animal toxicity), which confirmed the non-toxic nature of intermediates. Thus, the inclusive study of DMS2 showed promising efficiency in bioremediation approach for treating industrial effluents.

## Introduction

Azo dyes (R_1_-N=N-R_2_) are well known industrially synthesized organic compounds. The chemical structure of these dyes are easy to incorporate or replace its functional group, which make them highly versatile and stable in the environment (Balapure et al., 2014). During processing, textile and dyeing industries uses huge amount of water and such type of effluent contains about 10–40% of unused dyestuff which upon released into the environment causes serious pollution problem (Sahasrabudhe et al., 2014). Continuous disposal of such toxic dyestuff into the water bodies increases the organic load of natural reservoirs, which in turn leads to the negative impact on aquatic or terrestrial environments and its ecological functions (Saratale et al., 2015). Thus, it becomes essential to eliminate these compounds before it gets released into the environment (Das and Mishra, 2017).

Several conventional methods for treatment of textile dye effluents and polluted sites have been applied to date with limited success, alternatively microbial treatment methods are cost-effective and environmental-friendly (Agrawal et al., 2014; Govindwar et al., 2014). Microorganisms from contaminated environment adapt and modify themselves for the degradation of xenobiotic compounds exhibiting the prodigious catabolic activity toward the polluted environment (Hsueh et al., 2017). Therefore, the indigenous microorganisms are more favorably used for the development of bioremediation strategies. Many algae, fungi, actinomycetes and bacteria have been reported for decolorization and degradation of the azo dyes, among which bacterial cultures are economically favorable and shows promising results for the elimination of textile azo dyes effluents (Tan et al., 2013; Balapure et al., 2014; Kurade et al., 2016).

Biodegradation or decolorization efficiency depends on establishing the type of community adaption and providing the proper environment for the growth and activity of the enriched culture. Generally, reduction of azo compound occurs into the two phases; in first phase azo bond breaks by liberating the aromatic amine, which is comparatively more toxic than the parent dye (Balapure et al., 2014). While in the second phase, the mineralization of such aromatic compounds has been observed (Jayapal et al., 2018). Hence, to understand the feasibility of the bioremediation strategy in real-field applications, it is necessary to measure the toxicity of the metabolites produced after dye decolorization (Govindwar et al., 2014).

Direct Red 81 (DR81) is a diazo compound, having hydrophilic properties. Upon reduction of azo bonds under anaerobic or reducing environment, the corresponding aromatic compound may exert cytotoxic or genotoxic effect on microbial and human cells (Sahasrabudhe et al., 2014). In the present study, DR81 degrading pure culture of *Bacillus* sp. DMS2 was isolated from long-term azo dye polluted sites. To enhance the efficiency and understand the effect of one factor on another, the Plackett-Burman and response surface method (RSM) was performed for the optimization of nutritional and environmental parameters. The degradation pattern of DR81 was observed by enzymatic estimation and metabolic profiling using various analytical methods such as FTIR, LC-MS, NMR and UV-visible spectroscopy. To understand the efficacy of DMS2 and its potential in detoxification of textile dyes, toxicity assays were implemented using nematode and seed germination models.

## Materials and Methods

### Chemicals and Dye Stuff

Diazo sulfonated DR81 (CAS No.: 2610-11-9; Disodium 7-benzamido-4-hydroxy-3-[[4-[(4-sulphonatophenyl)azo] phenyl]azo] naphthalene-2-sulfonate) and other structurally different dyes were purchased from the local textile industry, Amardeep Dye industries, Vatva, Ahmedabad, Gujarat, India. All other chemicals used were of analytical grade purchased from Sigma (St. Louis, MO, United States), HiMedia, Merck and SRL (India).

### Bacterial Enrichment and Culture Conditions

Soil sediment was collected from Vapi industrial disposal site (20°22′N 72°54′E), Gujarat, India. Different dilutions of 10% soil sediment were plated on Bushnell Hass minimal medium amended with Glucose (2 g/l) and Beef extract (2 g/l) (BGB medium) along with DR81 (100 mg/l) at 37°C. Bacterial isolates with clear zone around colonies were screened for their dye decolorization ability. The strain with high decolorization capacity was used for azo-reduction analysis. The isolate was continuously sub-cultured into the fresh BGB medium amended with DR81 dye (100 mg/l) under microaerophilic condition and further maintained in BGB medium at 4°C.

### Identification and Phylogenetic Analysis of Bacterial Culture

The 16S rRNA gene sequence analysis was performed for the identification of strain DMS2. The bacterial culture was allowed to grow for 24 h. Genomic DNA of strain DMS2 was extracted using the previously described protocol (Ausubel et al., 1997). Universal primer 8F and 1492R were used for the 16S rRNA gene amplification. A 1.5 kb PCR amplicon product was purified and sequenced using automated DNA analyzer 3500 using BigDye^TM^ Terminator Cycle Sequencing v3.1 chemistry (Life Technologies, United States). Phylogenetic analysis was performed using MEGA 4.0 software (Shah et al., 2016).

### Azo-Reduction Experiment

Bacterium was grown in BGB (100 ml) medium amended with DR81 (100 mg/l) at 35°C for 24 h. Three milliliter of grown *Bacillus* sp. DMS2 was harvested at 7000 × g for 10 min at 4°C. The cell mass obtained were resuspended in sterile 1 ml distilled water and used as inoculum for further experiments, such as nutrient optimization study (by statistical model, as described in following sections), effect of various environmental conditions (NaCl concentration (0–100 g/l); DR81 concentration (100–2000 mg/l), 30 structurally different dyes as described above (100 mg/l each) on dye decolourization and degradation process under microaerophilic conditions at 35°C. The azo-reduction (decolourization) of DR81 was spectrophotometrically measured at 514 nm (Double Beam Specord^®^ 210 BU UV-Vis spectrophotometer, Analytical Jena AG, Germany). The abiotic control consists of uninoculated media containing 100 mg/l DR81. All the experiments were performed in triplicates.

The azo-reduction efficiency was calculated using an Equation 1 (Liu et al., 2017).

(1) %decolorization=(I-F)/I×100

where, I and F represent the absorbance at initial (I) and final (F) experiment, respectively.

### Statistical Designs for the Dye Decolorization

Media optimization for dye decolorization by DMS2 was carried out using the statistical design of experiments in two steps. In the first step, manageable set of variables were screened out by Plackett-Burman design and in the second step significant variables were optimized by employing central composite design (CCD). MINITAB v16 (Minitab Inc., State College, PA, United States) software was used for designing and analysis of the experiments.

#### Plackett-Burman Design

Total twenty medium components including carbon sources, organic-inorganic nitrogen sources, buffering agent and minerals were evaluated at higher concentration (+) and lower concentration (−) for dye decolorization (Supplementary Table S1). With respect to their main effects, Plackett-Burman design of 24 variables was selected for twenty media components with three dummy variables (Supplementary Table S2) and the dye decolorization response was measured (Plackett and Burman, 1946). The incorporation of dummy variables in an experiment is needed to estimate the experimental error. Highest positive influence showing variables from the Pareto chart analysis were considered to have a greatest impact on dye decolorization.

#### Central Composite Design (CCD) for Maximum Decolorization

Synchronizing the interaction between variables (statistical approach) may provide idea about the most favorable condition for the dye decolorization. Response surface methodology is a statistical approach used to design the experiments and evaluating the effect of variables by searching the optimum condition for specific responses. Here, CCD was performed for the maximum dye decolorization using MINITAB software. Significant and highly positive independent variables (five) from the Plackett–Burman experiment were selected for further optimization. Five individual variables i.e., MgSO_4_ (X1), glucose (X2), urea (X3), pH (X4) and temperature (X5) were selected for optimization. Combined effect of variables was studied (Supplementary Table S3). As per five factor design, *Bacillus* sp. DMS2 was observed for dye decolorization after 24 h of incubation. The independent variables were coded using Equation 2.

(2) xi=(Xi-Xo)/ΔXi

Here, Xi-variables: Xo-midpoint of Xi; ΔXi-change in Xi; and xi-code value of Xi; *i* = 1, 2, 3, 4 variables.

In this experiment, each constituent were considered with five levels, among which maximum values were coded as + 2 and the center point as zero. Forty-two combinations of five level and five center point replicate runs were performed. Here, central points were replicated to check the non-linear relationship between variables (Table 1).

**TABLE 1:** Estimated regression coefficients for percent decolorization.

Term	Coef SE	Coef	*T*	*P*
Constant	0.8162	3.329	0.245	0.808
MgSO_4_ (%)	1.1169	2.567	0.435	0.667
Glucose (%)	22.0557	2.567	8.591	0.000
Urea (%)	−1.7576	2.567	−0.685	0.500
pH	4.3434	2.541	1.709	0.099
Temperature	−49.5491	2.614	−18.956	0.000
MgSO_4_ (%)*MgSO_4_ (%)	54.1225	5.806	9.322	0.000
Glucose (%)*Glucose (%)	29.3170	5.806	5.049	0.000
Urea (%)*Urea (%)	13.2448	5.806	2.281	0.031
pH*pH	13.6402	5.264	2.591	0.015
Tem.*Tem.	56.5538	5.856	9.657	0.000
MgSO_4_ (%)*Glucose (%)	4.1571	5.387	0.772	0.447
MgSO_4_ (%)*Urea (%)	−4.8686	5.387	−0.904	0.374
MgSO_4_ (%)*pH	6.3523	4.818	1.318	0.199
MgSO_4_ (%)*Tem.	−3.2979	5.507	−0.599	0.554
Glucose (%)*Urea (%)	7.2733	5.387	1.350	0.189
Glucose (%)*pH	−0.1040	4.818	−0.022	0.983
Glucose (%)*Tem.	−30.2152	5.507	−5.487	0.000
Urea (%)*pH	−12.6753	4.818	−2.631	0.014
Urea (%)*Tem.	10.4294	5.507	1.894	0.069
pH*Tem.	3.8082	4.925	0.773	0.446

The optimal point can be predicted by understanding the relationship between individual variables (X1–X5 and Y-dye decolorization) using an Equation 3.

(3) Y=βo+ΣβiXi+ΣβijXi2+ΣβijXiXj

Here, Y-predicted dye decolorization; βo, βi, βii, βij-fixed regression coefficients of the model; Xi and Xj (*i* = 1, 2, 3, 4 and 5, i ≠ j, i < j = 1, 2, 3, 4, 5, 6) represent independent variables.

### Azo-Reduction in the Presence of Other Stress Compounds

DMS2 was studied for the azo-reduction in the presence of different NaCl concentration (0–100 g/l), initial dye concentration (50–2000 mg/l) and thirty structurally different dyes under optimized conditions.

### Enzymes Responsible for the Dye Decolorization

At regular time interval of azo-reduction, cell-mass was harvested by centrifuging at 8,000 × *g* for 15 min at 4°C. Cell free extract of DMS2 were prepared using previously published protocol (Balapure et al., 2014). Cell free lysate was further analyzed for the production of different enzymes like laccase, azo-reductase, NADH-DCIP reductase, tyrosinase and lignin peroxidase.

Laccase production was assessed as described in previous study (Hatvani and Mécs, 2001). Azo-reduction was performed by measuring the decrease in the absorbance of DR81 at 514 nm (Zimmermann et al., 1982). NADPH-DCIP, Tyrosinase and lignin peroxidase activity were estimated by using the previously reported protocols (Meng et al., 2012). The specific unit activity of the enzyme was defined as a change in absorbance/min/mg of enzyme protein under ambient conditions. Total protein was estimated by Folin-Lowry method (Lowry et al., 1951). All the experiments were performed in triplicates (*n* = 3).

### Azo-Reduction Pathway Analysis

DMS2 was allowed to grow in presence of DR81 (100 mg/l) in the optimized medium (Urea, Glucose and MgSO_4_) under microaerophilic condition at 30°C. Cell free supernatant was harvested after 8, 12, 24, and 48 h and centrifuged at 8,000 × *g* for 30 min. Cell free supernatant were concentrated using Rota evaporator (Bruker, United States), operated at 40°C and 100 rpm speed with 30 Pa of pressure. These concentrated metabolites were used for HRLC-MS, FTIR, NMR and UV-visible analysis.

LC-MS analysis was performed using Ultra-HPLC equipped with a PDA Detector-Mass spectrometer (Agilent Technologies, United States) and a C18 reverse phase column (5 μm, 250 × 4.6 mm). Methanol:water (60:40) was used as a mobile phase with the flow rate of 1.0 ml/min. The fractions from the liquid chromatogram were further analyzed by electron spray ionization (ESI) mass spectrometry (MS) using a LCQ Fleet Ion Trap LC/MS (Thermo Scientific, United States) (Nouren et al., 2017). Concentrated metabolites were mixed with FTIR grade potassium bromide (KBr) in the ratio of 3:97 (Sample:KBr) and analyzed from 600 to 4000 cm^–1^ with 20 scan speeds using FTIR spectrophotometer (Bruker, United States). Metabolites were further analyzed using 400 MHz of ^1^H and 100 MHz of ^13^C Advance-II FT-NMR spectrophotometer (Bruker, United States). To understand the degradation pattern, UV-visible scan from 200 to 800 cm^–1^ was performed by using Double Beam Specord^®^ 210 BU UV-Vis Spectrophotometer, Analytical Jena AG, Germany.

### Toxicity Analysis

#### Phytotoxic Analysis

Phytotoxicity study was performed to understand the toxic effect of DR81 and its metabolites produced after degradation by DMS2. The seeds of *Raphanus sativus, Vigna radiata* and *Abelmoschus esculentus* were used in the phytotoxicity assays because they are the common agricultural crops (Shah et al., 2016). The seeds were allowed to germinate in the presence of dye and metabolites. Plumule length and radical length were measured after 10 days of development. Simultaneously, experiment was carried out with distilled water under ambient conditions (Balapure et al., 2014).

Furthermore, *Lemna minor* (Duck weed) a model aquatic plant was cultivated under controlled condition (22°C, humidity-50RH and provided with white light) in 24 well plates (well volume of 5 ml, well surface area of 9.62 cm^2^; NUNC A/S, Denmark) with Steinberg medium (SM) (3 ml/well) provided with DR81 (100 mg/l) and metabolites (100 mg/l) (Zezulka et al., 2013). Plantlets were observed for germination, dry weight and content of photosynthetic pigments (chlorophyll-a and b). Reactive oxygen species (ROS) production was studied using a ROS sensing probe 2′,7′-Dichlorodihydrofluorescein diacetate (DCFH-DA) (Eugene, OR, United States) (Rastogi et al., 2010).

#### Effect of Dye and Metabolites on *Caenorhabditis elegans*

*Caenorhabditis elegans* N2 Bristol (Wild Type) obtained from the Caenorhabditis Genetics Center (CGC) at the University of Minnesota, Minneapolis MN, United States, which were grown at 20°C on Nematode growth medium (NGM). Experiment was carried out to study the worm synchronization and life assay of *C. elegans* seeded with *E. coli* OP50 under standard laboratory conditions and protocols (Sonani et al., 2014). The effect of dye and metabolites was confirmed on NGM plate supplemented with different concentrations ranging from 25 to 100 ppm. The number of dead worms was counted on every alternate day and the live worms were transferred to fresh NGM plates supplemented with or without metabolites and dyes of different concentrations. The mechanical stimulus and pharyngeal pumping rate were considered as substitute markers to record dead nematodes. The rhythmic convulsions of pharynx over 20 s were counted manually on 1st and 4th days to determine the rate of pharyngeal pumping (Sonani et al., 2014). To acquire the mean lifespan of treated and untreated worms, the plot of fraction survival against time (days) was constructed by taking treatment day as first day and subjected to log-rank test.

Locomotion assay was carried out under stereo-microscope by counting the number of body bends of 1st and 4th day adult worms during a 30 s interval. The altered reciprocating movement of mid-body bending in worms was defined as a body bend and experiment was performed with or without metabolites and dyes supplemented NGM with their respective concentration (Kumar et al., 2010).

### Statistical Analysis

All the data other than optimization were statistically analyzed using one-way analysis of variance (ANOVA) and Tukey-Kramer multiple comparison test.

## Results and Discussion

### Screening for Azo-Reduction

Isolation and screening of bacterial culture from polluted soil sample was carried out by culture enrichment technique using minimal medium (BGB) and DR81 (100 mg/l) as sole source of energy. Bacterial strain was selected on the basis of their ability to form larger clear zone indicating faster decolorization on DR81 containing BGB plate. Isolated bacteria was gram-positive, rod shaped, facultative anaerobic with flagella and produces white colored colonies. The 16S rRNA gene homology study revealed that bacterial strain DMS2 was closely related to the *Bacillus* sp. Hence, selected strain was identified as *Bacillus* sp. DMS2 (NCBI accession number MH201195) (Supplementary Figure S1).

**FIGURE 1:**
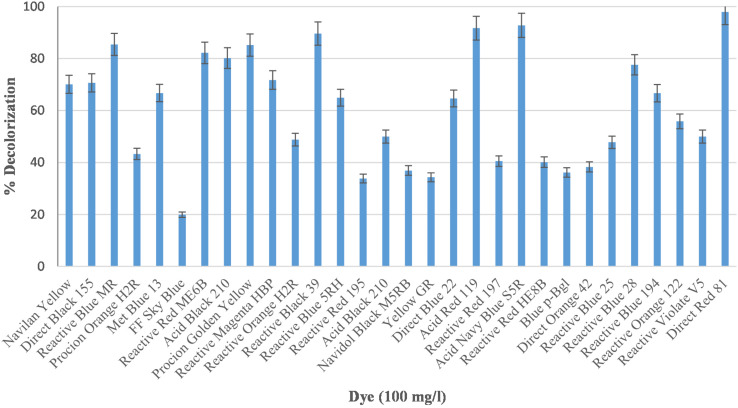
Decolorization efficiency of *Bacillus* sp. DMS2 toward 30 different azo dyes under optimized conditions.

Industrial effluents usually contain structurally diverse and different class of dyes of synthetic origin. We therefore, have examined *Bacillus* sp. DMS2 for its capability to decolorize 30 structurally different dyes. More than 90% of dye decolorization was observed with eleven different dyes among which DR81 showed highest decolorization, while 25% of other dyes showed >80% decolorization (Figure 1). There were only five dyes showing <30% decolorization. The dissimilar rate in dye decolourization by DMS2 can be attributed to the variation in chemical nature and structure of different dyes (Kurade et al., 2013; Pramanik and Chaudhuri, 2018). Based on the observed results, DR81 was selected as a model dye for further studies.

### Optimization of Nutritional and Environmental Parameters Using Statistical Parameters

The nutrient requirement and effect of environmental parameters on dye degradation was optimized using statistical methods. Plackett-Burman Design (PBD) predicts the significantly positive variables and Response surface method (RSM) predicts the interaction and the range of the independent variables. In the present study, effect of 20 different sources like carbon, nitrogen and minerals were evaluated for the dye decolorization using PBD (Supplementary Table S1). Significantly positive variables were selected for further optimization (Supplementary Figure S2). For the Central Composite Design (CCD) approach based on Response Surface Methodology was applied to five variables i.e., MgSO_4_, Glucose, Urea, pH and temperature. From the CCD, 47 runs with interaction of five independent variables were performed as shown in Supplementary Table S4. To determine the significance of the regression co-efficient with variables, student’s *T*-test and *P*-values were determined, which indicates the level of significance of parameters. The *P*-value of <0.05 indicate the high significance of the parameters. ANOVA analysis of the model revealed that this second order RSM model (Equation 3) was significantly fitted (*p* = 0.000) for the dye decolorization (Table 1).

Using MINITAB, for each run’s decolorization activity was predicted from the regression Equation 3 and accordingly experimental response was observed (Supplementary Table S4). ANOVA also showed that linear coefficients of glucose and urea were highly significant with the *P*-value of 0.000. The corresponding coefficient term is more significant when the magnitude of T is larger and *P*-value is smaller (Montgomery, 1991). Previous studies reported that lower the probability of Fisher value (*p* = 0.000) the higher significance for regression model (Kainthola et al., 2019). In the model, the determination coefficient *R*^2^ = 96.48% and adjusted determination coefficient *R*^2^ = 93.77% shows the significant performance of the model.

**FIGURE 2:**
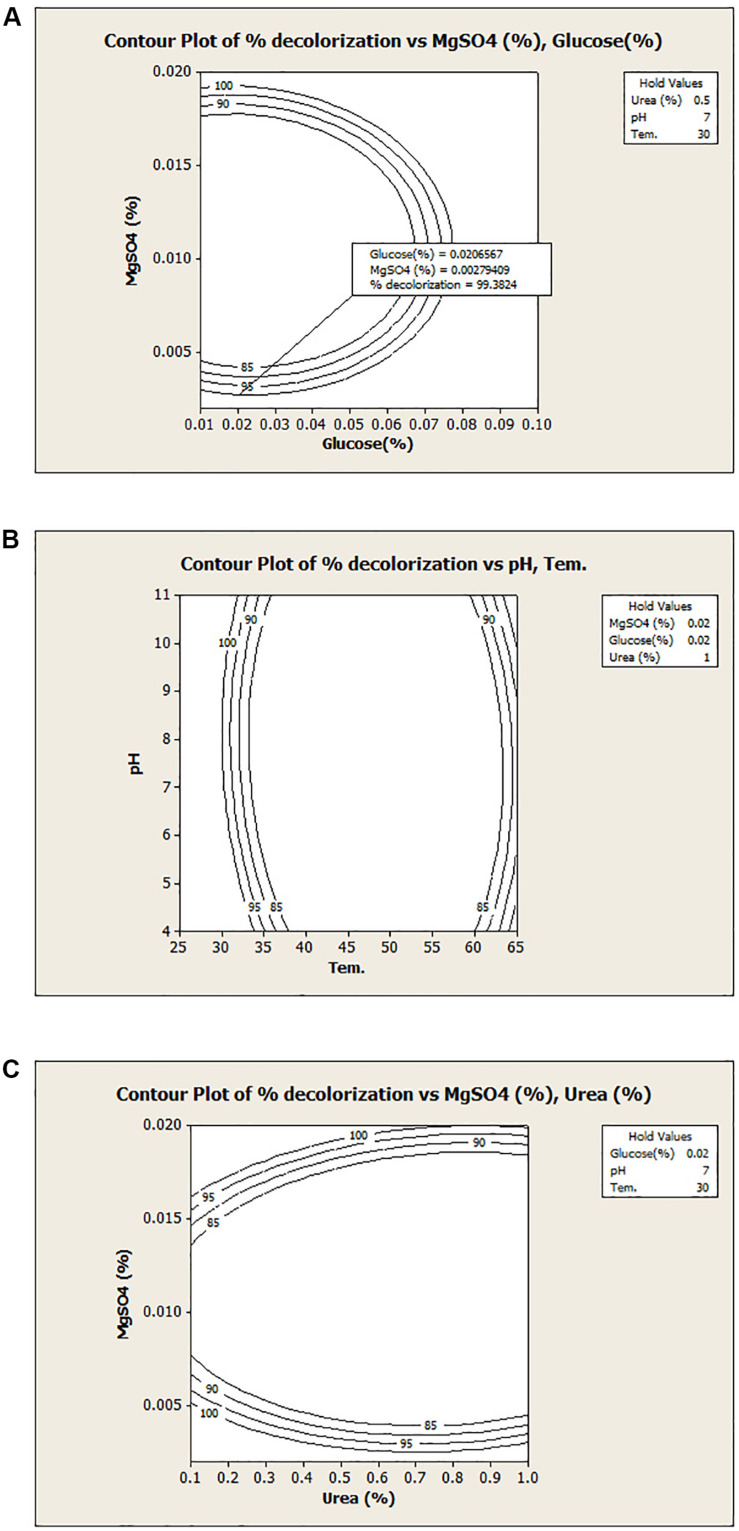
Contour plots of the decolorization with different variables: **(A)** MgSO_4_ *v/s* Glucose; **(B)** temperature *v/s* pH and **(C)** MgSO_4_ *v/s* urea.

A graphical presentation of the model shows the interaction between the independent test variables. Figure 2A illustrates the relationship between glucose and MgSO_4_ for the dye decolorization at 30°C under microaerophilic condition. Counter plot of the model shows that the decolorization activity was decreased at middle value of MgSO_4_ with both nutritional sources (glucose and urea) and at low temperature range, decolorization increased, irrespective of pH range (Figures 2B,C). Hence, from the model, it has been revealed that MgSO_4_ and temperature play a critical role in dye decolorization and organism is highly stable under alkaline or acidic conditions. Here, from this model, results suggested that interaction between glucose and pH, MgSO_4_ and temperature, MgSO_4_ and urea were not significant. Asserts that the decolorization efficiency of newly isolated bacterial culture *Bacillus* sp. DMS2 remain stable with wide range of pH. In a similar previous study, a dye decolorizing, pH stable *Acinetobacter* sp. and *Klebsiella* sp. was isolated from the dye contaminated wastewater (Meerbergen et al., 2018). Azo dyes contain electro-deficient functional groups like –SO_3_, which make dye less susceptible to degradation. Hence, co-substrates along with dye (carbon and nitrogen source) are needed for the growth and decolorization (Balapure et al., 2014).

#### Validation of Model

To validate the model, experiment was carried out under optimized conditions i.e., Glucose (0.02%) MgSO_4_ (0.002%), Urea (0.5%) at pH-7.0 and 30°C for 24 h under microaerophilic conditions. About 98.62% decolorization was observed under optimized conditions, while the predicted value from the model was 99.38%, which was in similar range that of experimental value. It was observed that optimization using the statistical approach has enhanced the dye decolorization capacity of *Bacillus* sp. DMS2 by nearly two-fold. After optimization, biodegradation time of DR81 decreased as compared to the initial experiment. Similarly, in another study app. 1.5 fold increase was noted in Amido black 10B decolorization after statistical optimization using *Kocuria kristinae* RC3 (Uppala et al., 2019).

### Decolorization Assessment in the Presence of Other Environmental Parameters

Salts are essentially required in dye and textile industries and their effluent might contain high salt concentration (i.e., ∼2000 to 3000 ppm), which may go up-to 15–20% (Guo et al., 2020). High salt and initial dye concentration decrease the cell metabolic activity, which might reduce dye decolorization rate of the bacterium (Balapure et al., 2014). Hence, the bacterial system for dye remediation must tolerate high salt concentration (Saratale et al., 2011). Supplementary Figure S3A shows that *Bacillus* sp. DMS2 has a capacity to tolerate up to 50 g/l of NaCl concentration and simultaneously decolorize 97% of DR81. Previous study has shown the halophilic alkalithermophilic bacterial consortium having the capacity to tolerate up to 10% of the NaCl (Guo et al., 2020). While in this study, upon increasing NaCl concentration >50 g/l, the decolorization capacity of DMS2 significantly decreased. Similar type of results were observed previously, using *Scheffersomyces spartinae* TLHS-SF1 for decolorization of Acid Scarlet 3R (20 mg/l) with initial salt concentration 30 g/l (Tan et al., 2016).

**FIGURE 3:**
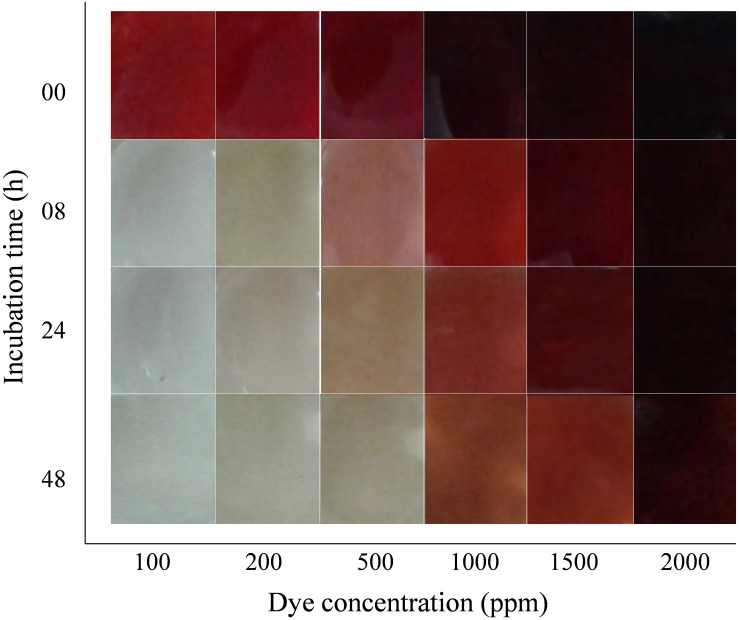
Effect of initial dye concentration on DMS2 decolorization capacity.

Initial dye concentration is inversely proportional to the decolorization capacity of the organisms. The decolorization capacity of the DMS2 at different initial dye concentration was pictorially measured. DMS2 was allowed to decolorize different dye concentrations and results were captured at different time intervals as shown in Figure 3. The observed results showed that DMS2 has decolorized 1000 mg/l of DR81 within 24 h. While with 1500 mg/l of DR81, DMS2 required 32 h for 80% decolorization (Supplementary Figure S3B). In our study, the decrease in efficiency was observed due to high toxicity of dye and the metabolites produced after degradation (Tan et al., 2016). Also, an earlier research described the linear increase in decolorization with initial dye concentration up to certain extent and which eventually reached the steady state due to toxicity (Guo et al., 2020).

### Assessing the Catabolic Efficiency of DMS2

A number of microbial enzymes have been reported for dye decolorization and degradation processes. For the biotransformation of xenobiotic compounds, cascade of various oxido-reductive enzymes play an important role (Saratale et al., 2013). To understand the decolorization and degradation mechanism of DMS2, enzyme activity of azoreductase, laccase, NADH-DCIP, lignin peroxidase and tyrosinase were studied (Kurade et al., 2016; Liu, 2017). Azo-bonds of the dye were broken down via azoreductase enzymes and generate aromatic amines (Singh et al., 2015; Zahran et al., 2019). Consequently, these amines undergo further cleavage by the use of different oxidative enzymes, which finally enter into the central metabolic pathways. DMS2 shows the significant induction of laccase (2.32 ± 0.11) and azoreductase (0.67 ± 0.001) compared to lignin peroxidase (0.05 ± 0.001), NADH-DCIP (0.1241 ± 0.003) and tyrosinase (0.07 ± 0.000) for the complete decolorization of DR81 (Table 2). Profiling of the catalytic action revealed the synergistic action of oxido-reductive enzymes for the total mineralization of DR81. Similar results were reported previously in which the induction of laccase activities were observed in *Bacillus stratosphericus* SCA1007 for methyl orange mineralization (Akansha et al., 2019).

**TABLE 2:** Estimation of enzymes responsible for dye decolorization and degradation.

Enzymes	0 h	12 h	24 h	32 h	48 h
Laccase^*a*^	0.03 ± 0.09	0.84 ± 0.01	2.32 ± 0.11	1.79 ± 0.06	0.42 ± 0.01
Azoreductase^*b*^	0.01 ± 0.02	0.31 ± 0.01	0.67 ± 0.05	0.45 ± 0.02	0.19 ± 0.04
Lignin peroxidase^*a*^	0.04 ± 0.03	0.05 ± 0.05	0.05 ± 0.04	0.05 ± 0.06	0.05 ± 0.01
NADH-DCIP^*c*^	0.04 ± 0.06	0.07 ± 0.03	0.12 ± 0.03	0.08 ± 0.05	0.05 ± 0.04
Tyrosinase^*a*^	0.01 ± 0.06	0.06 ± 0.02	0.07 ± 0.05	0.06 ± 0.02	0.04 ± 0.03

*Mean of three experiments ± SEM. Significantly difference form control cell at p < 0.05 and p < 0.01 by one way ANOVA with Turkey-Kramer comparison test. ^a^ Enzyme activity in unit/min/mg protein. ^b^ Enzyme activity in μM Methyl red reduced/min/mg protein. ^c^ Enzyme activity in μg DCIP reduced/min/mg protein.*

### Metabolites Estimation

To unveil the decolorization and degradation profiles of DR81, UV-visible, FTIR, LC-MS, ^13^C- and ^1^H-NMR was performed. UV-vis spectra (300–800 nm) of the dye showed its absorption maximum at 514 nm. Decrease in absorption of the culture metabolites supernatant withdrawn at different time intervals indicated the decolorization and decrease in concentration of DR81. The complete decolorization of the dye was observed within 8 h (Supplementary Figure S4). Disappearance of peak obtained at 514 nm indicated a complete decolorization of the DR81 dye. The result obtained here is in agreement with previous reports (Sahasrabudhe et al., 2014; Singh et al., 2015).

The degradation pattern of DR81 was studied using FTIR at 400–4000 cm^–1^ (mid-IR region). The IR spectra of pure DR81 showed the characteristic peaks of the dye compounds, where –N=N– stretching azo group was exhibited between 1500 and 1600 cm^–1^. The peak at 1656 cm^–1^ confirms the –C=N– stretching and –NH bending of secondary aromatic amines. The aromatic rings in the dye molecules showed characteristic peaks at 849, 714, 701, 638, and 610 cm^–1^. The meta-substituted –SO_3_ showed peak at 1387 cm^–1^. The –C-H stretching of unsaturated –CH_2_ exhibited peaks at 2959 and 2855 cm^–1^ (Supplementary Figure S5; Balapure et al., 2014). The ^1^H-NMR spectrum showed the characteristic downfield signal between δ 6 and 8 and intense signal at δ 7.45 of naphthalene ring of intact dye molecule. The signal at δ 2.2 showed the presence of amide group of dye compound. The ^13^C-NMR also showed the characteristic peaks of aromatic carbons and of other functional groups of dye molecules.

During decolorization and degradation of DR81 after 12 h, the IR spectra of the peaks of aromatic rings between 600 and 900 cm^–1^ were observed but with the lower intensity as that of intact dye compound. The specific peak at 619 cm^–1^ confirms the formation of corresponding aromatic amines. The peak at 1114 cm^–1^ indicates the overlapping of two functional group i.e., –SO_3_ and primary –NH_2_. While during further degradation, peaks for aromatic compounds disappeared. The peak at 1082 cm^–1^ confirmed the presence of bonded –OH group in the degraded intermediate and peak at 1656 cm^–1^ showed the –C=C– alkyl group. Asymmetrical vibration of free –S=O showed peak at 1082 cm^–1^. Another overlapping peaks for presence of bonded –OH and primary amine showed peak at 1080 cm^–1^. The absence of peak at 1191 cm^–1^ after 24 h indicates the complete disappearance of –S = O group. In ^1^H-NMR and ^13^C-NMR spectra, the intensity of peaks between δ 6–8 and δ 105–155 decreases with the progress in the degradation of DR81 (Supplementary Figure S6). In ^13^C-NMR at different stages of dye degradation, peaks at δ 129 showed the presence of substituted alkenes, peaks between δ 68 and 71 showed the presence of alcohols/ethers including nitro-group in the compound –CNO_2_. While the intermediate containing carboxylic acid group and ketone group were detected through ^13^C-NMR. Further, LC-MS analysis also illustrates the presence of non-toxic degraded metabolite compounds of the dye.

Through all the spectral analysis of DR81 degraded products at different time intervals revealed that at the initial stage, catabolic activity of DMS2 showed the increased azoreductase activity which is responsible for initial cleavage of diazo bond. This toxic catalytic products where further neutralized by the increased activity of the laccase enzyme present in *Bacillus* sp. DMS2. Hence, the synergistic effect of azoreductase and laccase resulted the formation of three intermediate compounds namely, 4-Hydroxystyrene (m/z, 103), 2-Napthaleneacetic acid-6-hydroxy (m/z, 120) and N,N-Dimethylaniline N-oxide (m/z, 185). The results from above study suggested that DR81 was symmetrically cleaved by DMS2. Hence, from all spectral analysis method described above the possible degradation pathway of DR81 was deduced and is illustrated in Supplementary Figure S7. There are number of reports which show similar proposed pathways in bacterial system on the basis of spectral analysis (Balapure et al., 2014; Shah et al., 2016).

### Toxicity Analysis

Several reports have shown that the toxicity of metabolites formed after dye degradation is comparatively higher than its parent dye compounds (Misal and Gawai, 2018; Saini et al., 2018). So, to verify the acceptance of degradation, it is important to measure the toxicity response of parent dye and its degraded products. The pollutants and degraded metabolites have highly toxic effects on aquatic and terrestrial environment; hence, we conducted phytotoxicity study on both environments. Furthermore, we also tested the toxicity of dye and metabolites on nematode model *C. elegans*.

#### Terrestrial Phytotoxicity

In the presence of dye DR81, the reduced germination of *V. radiata*, *R. sativus* and *A. esculentus* seeds was observed with only 30, 30, and 36% of seeds germination, respectively. After decolorization/degradation of dye, the germination extended further to more than 90% in presence of metabolites. We measured plant’s photosynthesis apparatus, root length and shoot length to validate our results (Table 3). In comparison to dye, better growth of experimental plants was observed when grown under dye degraded products. The previous studies using *Phaseolus mungo* and *Vigna radiata* to understand the toxic effects of the dye also revealed the non-toxic nature of degraded products after decolorization (Bedekar et al., 2015; Shah et al., 2016).

**TABLE 3:** Phytotoxicity analysis of DR81 (100 mg/l) and metabolites produced after decolorization.

	*V. radiate*			*R. sativus*			*A. esculentus*		
	Control	Dye	Product	Control	Dye	Product	Control	Dye	Product
Germination (%)	99.00 ± 1.00	30.00 ± 2.00	96.60 ± 1.52	99.66 ± 0.57	30.00 ± 2.64	940.0 ± 1.00	99.33 ± 1.15	36.33 ± 1.15	94.66 ± 1.52
Root length (cm)	9.18 ± 0.02	4.25 ± 0.32	8.56 ± 0.30	8.13 ± 0.20	4.13 ± 0.20	7.19 ± 0.13	12.76 ± 0.28	6.30 ± 0.26	10.33 ± 0.41
Shoot length (cm)	7.30 ± 0.26	2.16 ± 0.12	6.60 ± 0.30	8.31 ± 0.25	5.11 ± 0.08	6.15 ± 0.29	7.23 ± 0.09	2.06 ± 0.12	5.95 ± 0.10
Biomass (g)	0.30 ± 0.07	0.09 ± 0.01	0.27 ± 0.09	0.20 ± 0.03	0.07 ± 0.06	0.19 ± 0.03	0.41 ± 0.07	0.19 ± 0.02	0.39 ± 0.06
Chlorophyll-a (μ g/ml)	11.26 ± 0.37	8.14 ± 0.22	10.24 ± 0.62	12.13 ± 0.22	8.58 ± 0.52	11.41 ± 0.39	11.98 ± 0.07	8.29 ± 0.34	11.13 ± 0.13
Chlorophyll-b (μ g/ml)	4.54 ± 0.35	2.50 ± 0.39	4.12 ± 0.22	4.34 ± 0.21	2.77 ± 0.34	4.19 ± 0.22	5.10 ± 0.16	2.30 ± 0.13	4.67 ± 0.38
Carotenoids (μ g/ml)	8.49 ± 0.93	3.56 ± 0.17	8.26 ± 0.08	11.15 ± 1.78	3.72 ± 0.46	8.25 ± 0.46	10.22 ± 0.92	3.97 ± 0.31	7.09 ± 0.89

#### Aquatic Phytotoxicity

**FIGURE 4:**
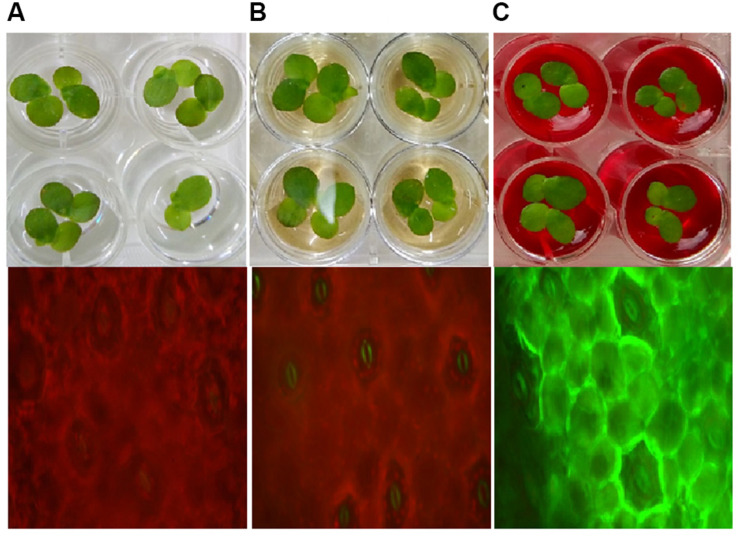
*In vivo* ROS production in *L. minor* seed germination with **(A)** control sample; **(B)** dye degraded products and **(C)** dye.

**FIGURE 5:**
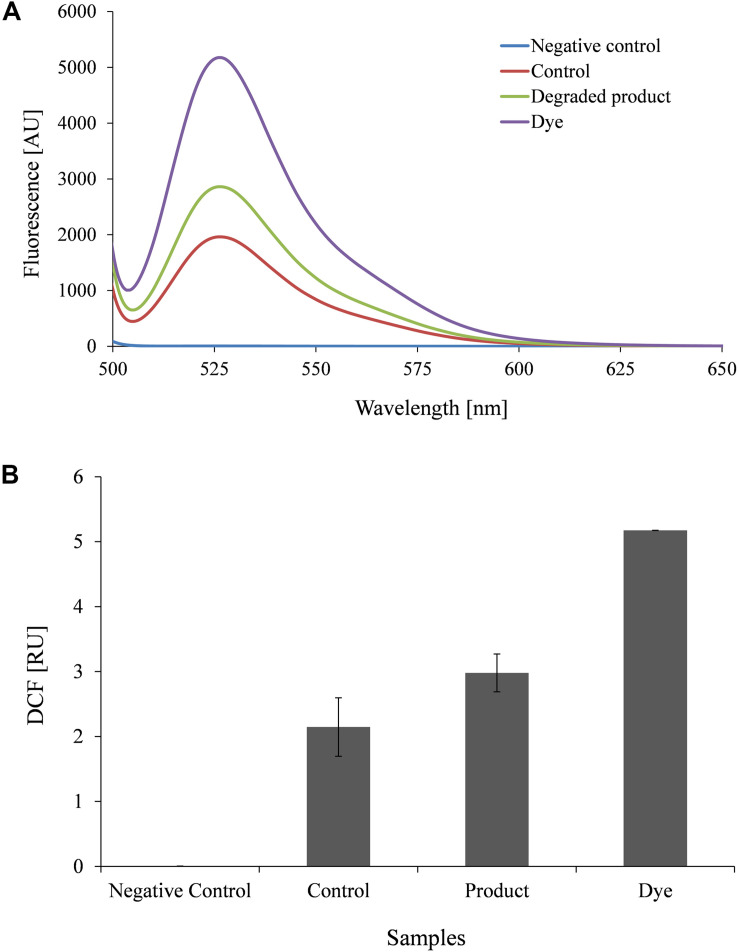
Fluorophotometric analysis of *in vitro* ROS production in *L. minor* growing with dye and degraded dye products **(A)** Emission spectra of DCF fluorescence **(B)** DCF fluorescence intensity.

To understand toxicity of dye and its degraded products, we conducted the aqua-phytotoxicity assay on a model aquatic plant *L. minor*. After 10 d of incubation in the presence of dye, 90% growth inhibition was measured, while only 10% of growth inhibition was observed in presence of metabolites (Supplementary Table S5). To validate the results, physiological parameters such as *in vivo* and *in vitro* production of ROS in was measured in *L. minor* (Figures 4, 5). DCFH dye was non-fluorescent, but become highly fluorescent (green colored) when oxidized into DCF at the time of ROS production. Figure 5 represents the *in vitro* production of ROS in *L. minor* in the presence of dye and degraded products. In comparison to control and degraded product, treated samples, maximum ROS production was observed in dye-treated samples (Figures 4, 5). High production of ROS and reduction in chlorophyll level in the presence of dye was observed, indicating its toxic effects on plants of aquatic habitat (Souza et al., 2019; Sun et al., 2019). The levels of ROS and reduction of chlorophyll level was less in presence of degraded metabolites of DR81 (Supplementary Table S5; Karuppanapandian et al., 2011). A number of previous reports have confirmed the ROS production in plants in presence of some hazardous pollutants (Radić et al., 2018; Sun et al., 2019).

#### Effect of Dye on the Lifespan of *C. elegans*

**FIGURE 6:**
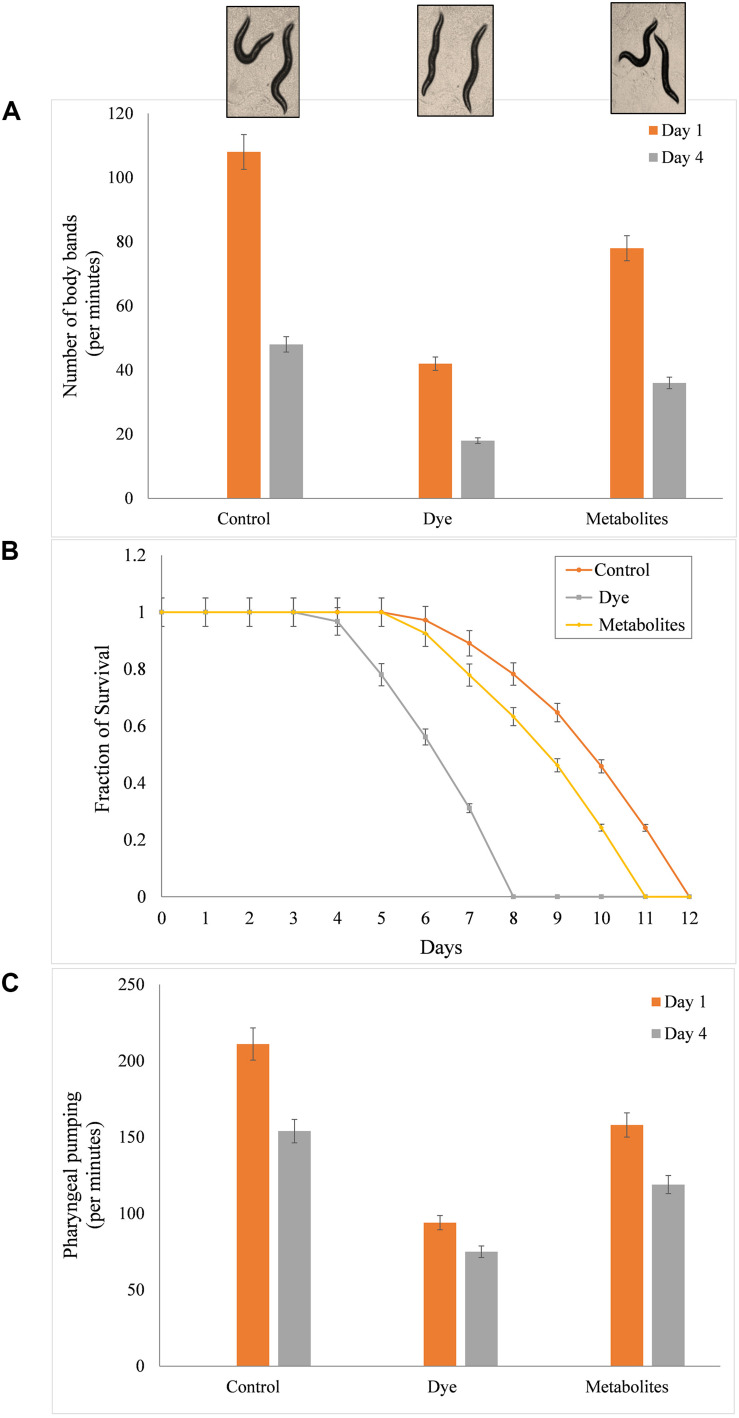
**(A)** Mean survival time of *C. elegans* with dye (100 mg/l) and metabolites produced after incubation with DMS2, **(B)** Toxicity analysis on the life span of *C. elegans* and **(C)** Effect on pharyngeal pumping.

The effect of dye and metabolites on lifespan of *C. elegans* was studied by growing L4 stage worms at 20°C under varying concentration (0, 25, 50, and 100 ppm) of metabolites and dye. Figure 6 shows the culturing of worms under control (without dye), in presence of dye and product of treated sample. The mean lifespan of control nematodes was found to be 12 ± 0.1 (mean lifespan ± standard error mean) days. While, metabolites and dyes treated nematodes showed values of 11 ± 0.1 days and 8 ± 0.2 days for 50 ppm, respectively (*p* > 0.05, log-rank test) (Figure 6B). Metabolites treated worms showed significant increase in longevity in comparison to dye treated worms. To determine the potency of metabolite in reducing the toxic effect of dye and its effect on the longevity of worms, we counted substitute physiological indicators of aging like rate of worm locomotion and pharyngeal pumping rate in metabolites treated and dye treated worms in time course of aging (1st and 4th day of post adulthood) (Figures 6A,C). The rate of pharyngeal pumping for control worms were found to 199 ± 1.05/min and 145 ± 1.38/min on 1st and 4th day, whereas metabolites and dye treated worms showed the rate of pharyngeal pumping upto 158 ± 3.5 and 119 ± 2.94/min, 94 ± 3.18 and 75 ± 2.83/min, respectively (Figure 6C). The locomotory behavior of metabolites and dye treated worms were assayed by counting the number of body bends per minute. The number of body bends per minute were 108 ± 0.31 (*p* < 0.0001, *t*-test) and 48 ± 0.40 (*p* < 0.0001, *t*-test) for control worm on 1st and 4th days, respectively. In compared to control, metabolites treated and day treated worms showed the body bends (per minute) upto 35.4 ± 0.40 (*p* < 0.0001, *t*-test) and 25 ± 1.61 (*p* < 0.0001, *t*-test), whereas 42 ± 0.50 (*p* < 0.0001, *t*-test) and 18 ± 0.31 (*p* < 0.0001, *t*-test) on 1st day and 4th day, respectively (Figure 6A). Metabolites treated worms showed significant improvement in pharyngeal pumping rate and locomotory response compared to control worms. Results suggested that metabolites treated worms showed attenuation in the aging associated declining in physiological functions such as feeding and locomotion to a certain extent. Henceforth, the degraded products are less of non-toxic for the both animal and plant systems, which proves that the newly isolated organism *Bacillus* sp. DMS2 is feasible for the filed application. This study was restricted up-to the laboratory level, however, we are working on scale up and then we will go for the *in situ* application.

## Technical Challenges and Future Research Perspectives

The real technical challenge for this study is to apply such bioremediation strategy *in situ* field. Further, observations in this research must be studied and verified for large scale industrial applications.

## Conclusion

There are very few reports which show a complete metabolism and toxicity reduction of the diazo dye compounds by a single bacterium. This study provides a complete understanding of the mechanism of textile diazo dye degradation by *Bacillus* sp. DMS2 along with the plausible interpretation of the dye degradation and metabolites formed after bacterial treatment. The experimental results revealed that *Bacillus* sp. DMS2 was not only effective in the decolourization of many textile dyes tested, but also simultaneously reduced the toxicity of degraded intermediates and parent dye compound under microaerophilic conditions. Results, obtained in this study indicate that the bacterium *Bacillus* sp. DMS2 can effectively decolourize and degrade the textile diazo dyes with low nutrient supplements and under ambient conditions.

Moreover, the treatment of DR81 dye by the bacterium showed significant reduction in its toxicity on plant and animal models. Therefore, *Bacillus* sp. DMS2 can be used as an efficient, cost-effective and eco-friendly bioremediation agent in bio-reactors treating textile wastewaters or for bioaugmentation of textile dye polluted soils to achieve biodegradation and reduction in toxicity of textile dyes.

## Data Availability Statement

The datasets generated for this study can be found in the *Bacillus* sp. strain DMS2 (Accession number MH201195).

## Author Contributions

SA performed the dye decolonization, degradation, as well as toxicity experimentation along with RR. MC performed experiments of *C. elegans*. JD helped in designing the experiments for RSM and interpreting its results. SA and KJ drafted the initial manuscript. RR and CD edited and refined the manuscript. KJ and DM provided the initial concept. SA developed the further objectives. All the authors read and approved the manuscript.

## Conflict of Interest

The authors declare that the research was conducted in the absence of any commercial or financial relationships that could be construed as a potential conflict of interest.

## Supplementary Material

FIGURE S1 Phylogenetic analysis of *Bacillus* sp. DMS2.

FIGURE S2 Pareto chart for the effects of variables.

FIGURE S3 Effect of DMS2 on NaCl concentration **(A)** and initial dye concentration **(B)**.

FIGURE S4 UV-vis overlay spectra at different interval during decolorization of DR81 by *Bacillus* sp. DMS2 under optimized conditions.

FIGURE S5 FTIR spectrum analysis of DR81 and its products after degradation by *Bacillus* sp. DMS2 at different time interval.

FIGURE S6 H^1^-NMR spectrum of DR81 and their degraded products by DMS2 at various time interval.

FIGURE S7 Proposed degradation pathway analysis of DR81 by consortium DMS2.
