# Supplementary figures and images for: Degradation and Toxicity Analysis of a Reactive Textile Diazo Dye-Direct Red 81 by Newly Isolated Bacillus sp. DMS2
Source: Front Microbiol. 2020 Sep 24;11:576680. doi: 10.3389/fmicb.2020.576680 (PMC7541843; doi:10.3389/fmicb.2020.576680)

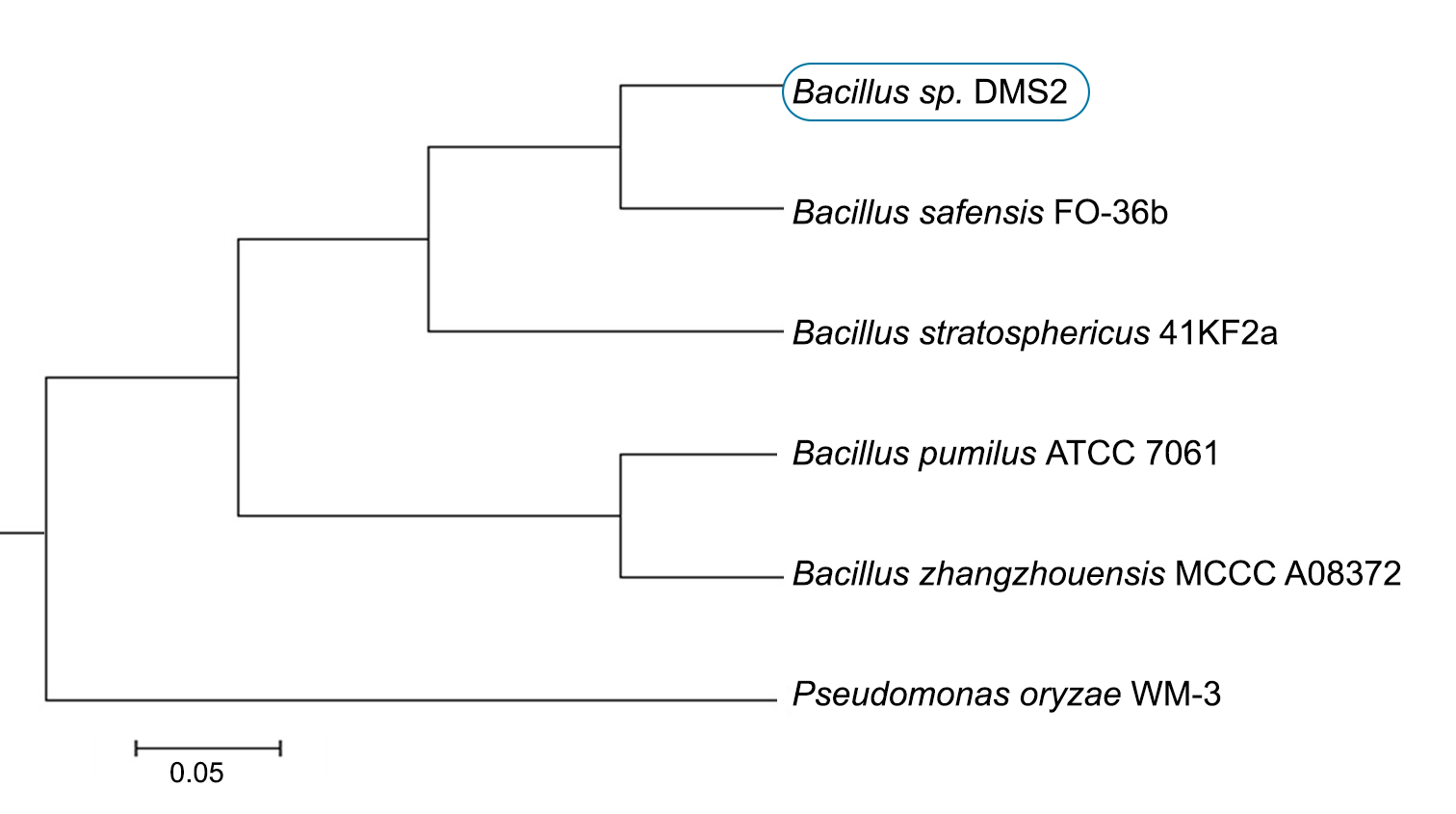

Supplement: FIGURE S1 — Phylogenetic analysis of Bacillus sp. DMS2. [file Image_1.JPEG]

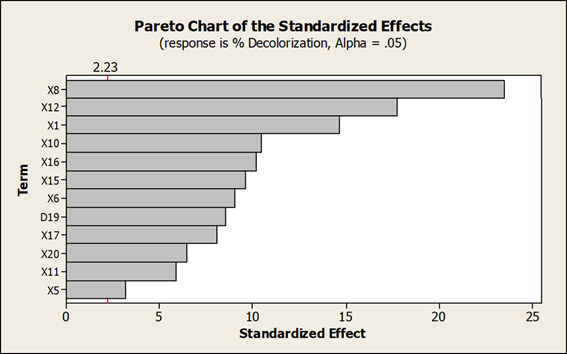

Supplement: FIGURE S2 — Pareto chart for the effects of variables. [file Image_2.JPEG]

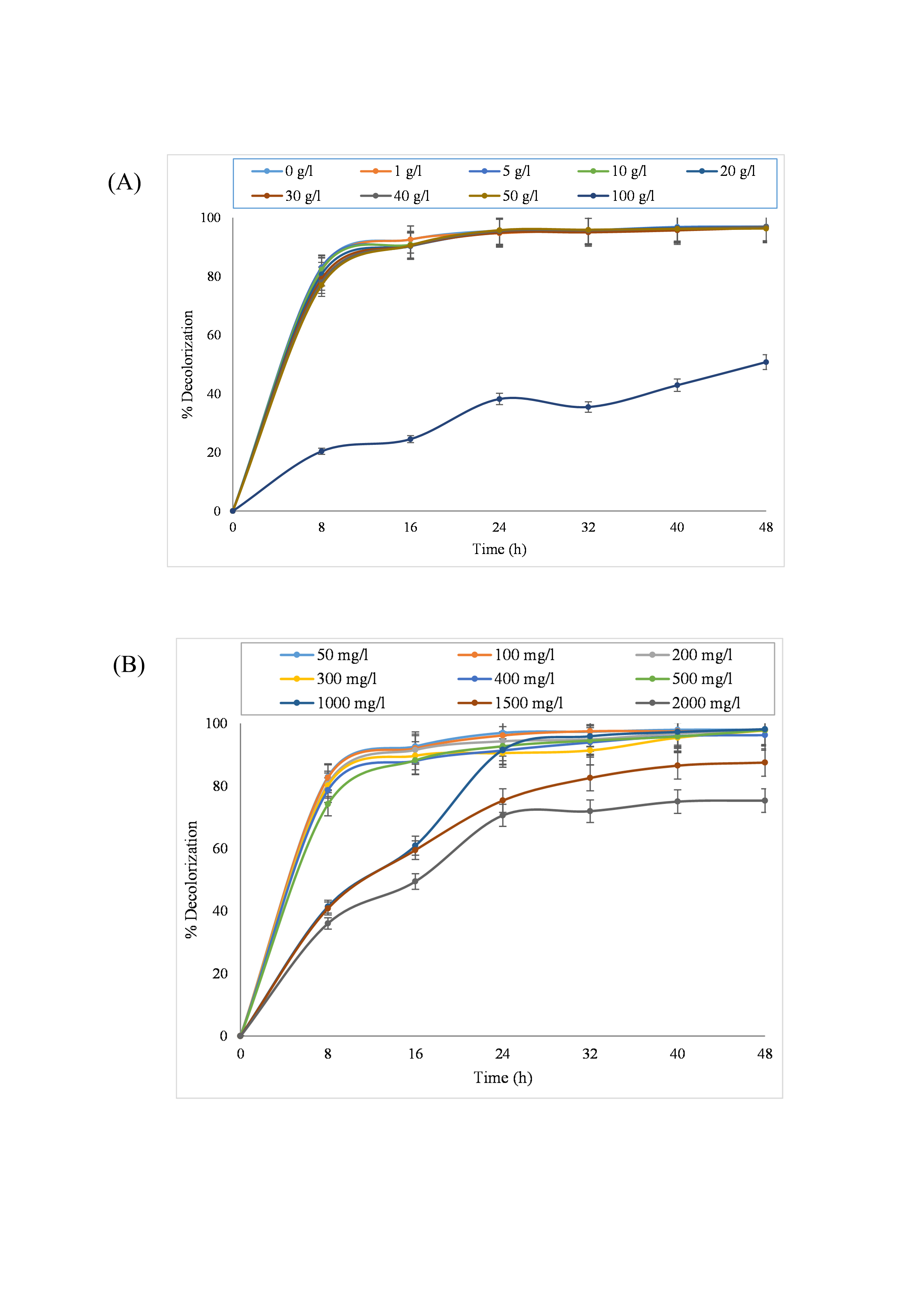

Supplement: FIGURE S3 — Effect of DMS2 on NaCl concentration (A) and initial dye concentration (B). [file Image_3.JPEG]

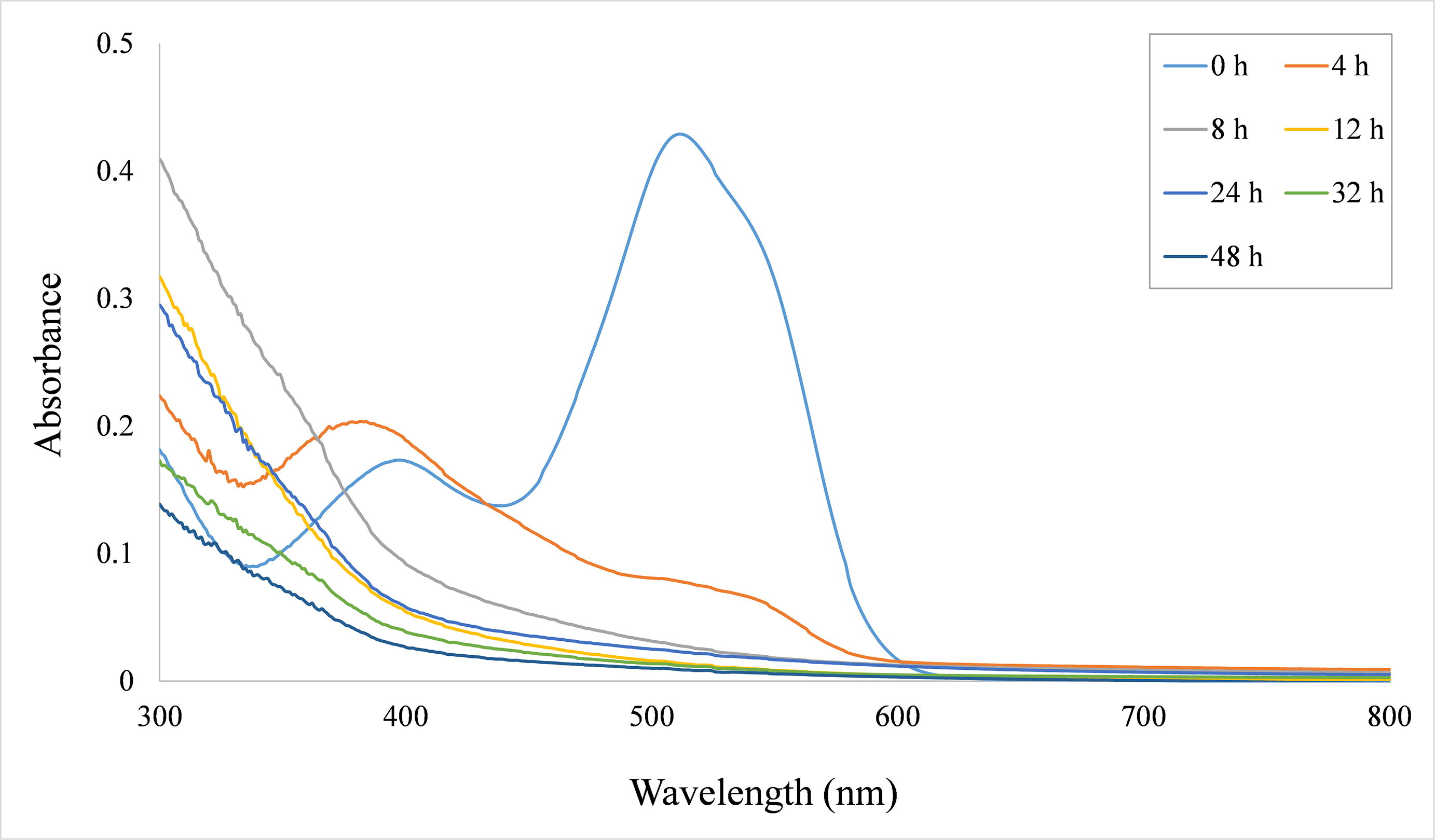

Supplement: FIGURE S4 — UV-vis overlay spectra at different interval during decolorization of DR81 by Bacillus sp. DMS2 under optimized conditions. [file Image_4.JPEG]

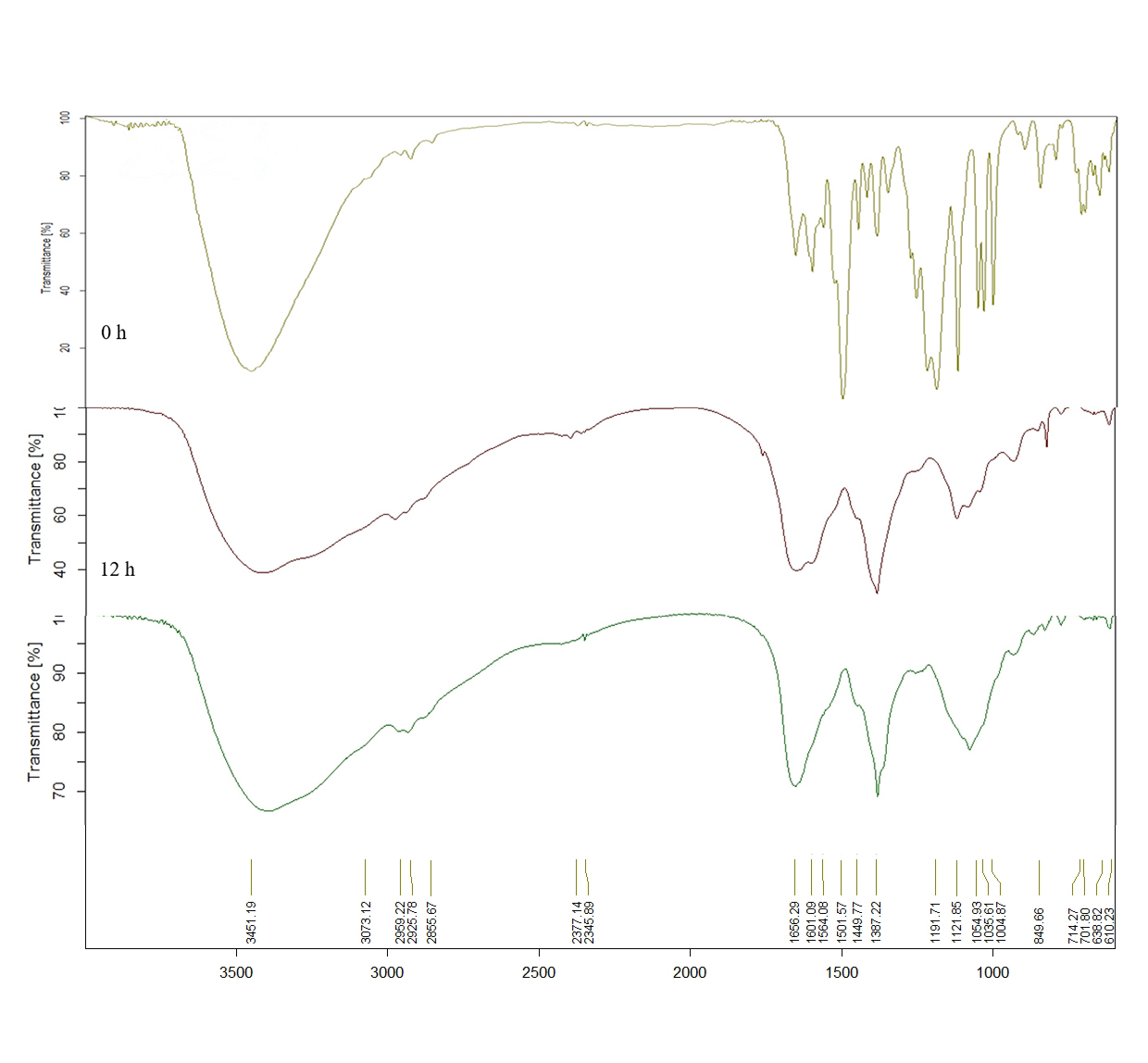

Supplement: FIGURE S5 — FTIR spectrum analysis of DR81 and its products after degradation by Bacillus sp. DMS2 at different time interval. [file Image_5.JPEG]

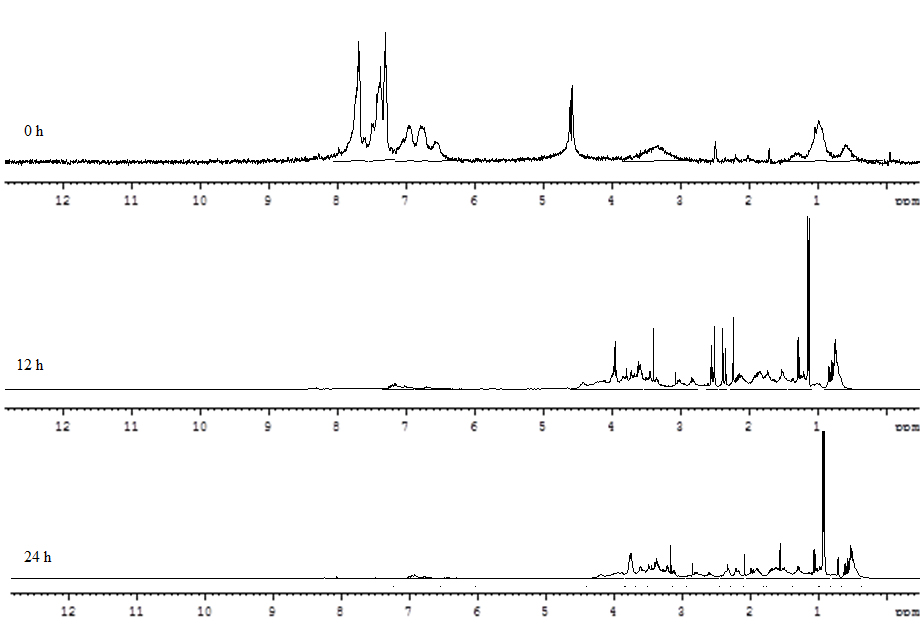

Supplement: FIGURE S6 — H1-NMR spectrum of DR81 and their degraded products by DMS2 at various time interval. [file Image_6.JPEG]

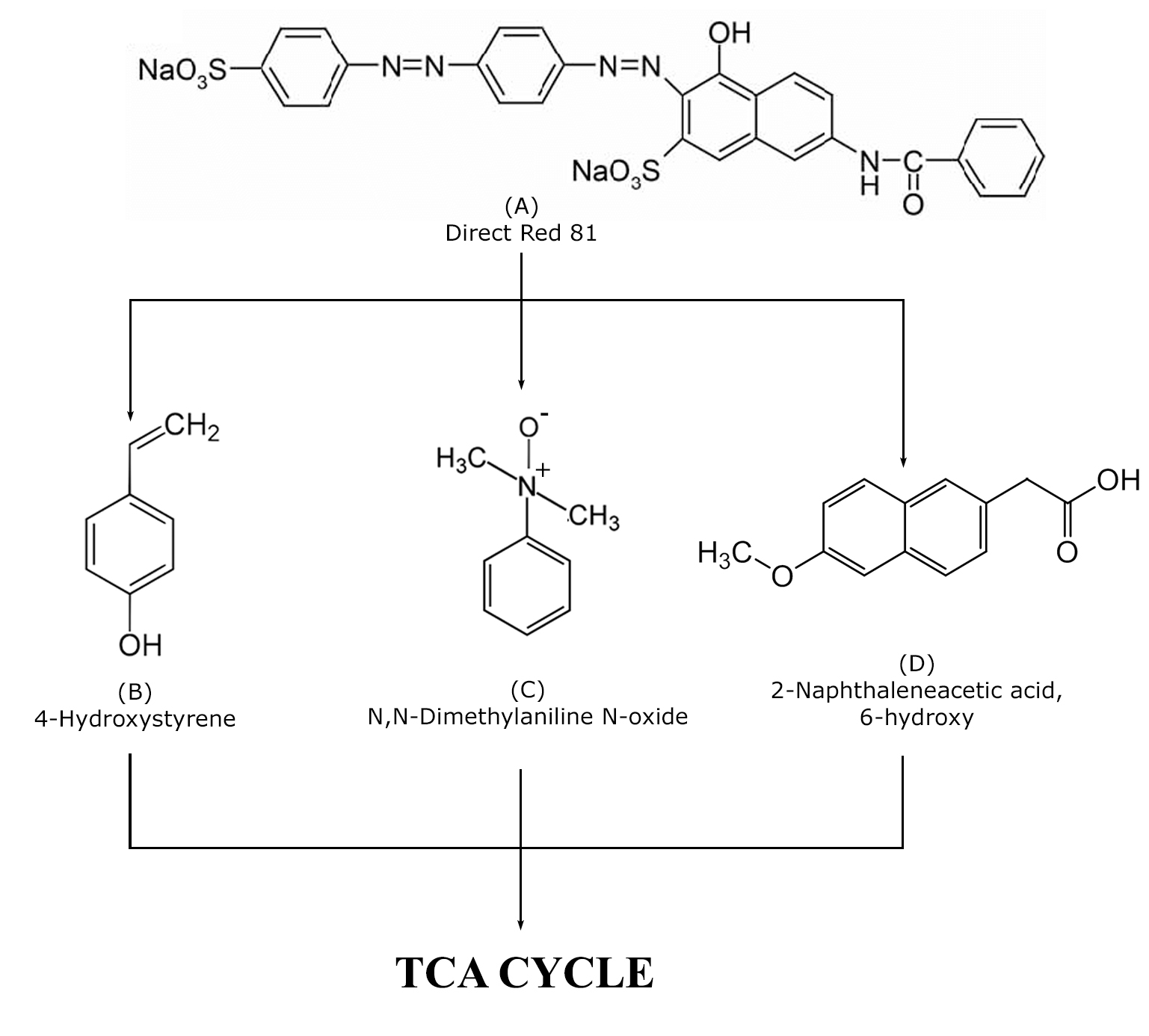

Supplement: FIGURE S7 — Proposed degradation pathway analysis of DR81 by consortium DMS2. [file Image_7.JPEG]
