# Supplementary material for: Degradation and Toxicity Analysis of a Reactive Textile Diazo Dye-Direct Red 81 by Newly Isolated Bacillus sp. DMS2
Source: Front Microbiol. 2020 Sep 24;11:576680. doi: 10.3389/fmicb.2020.576680 (PMC7541843; doi:10.3389/fmicb.2020.576680)
Supplement: Supplementary file 8 [file Data_Sheet_1.docx]

**Supplementary Data**

**Degradation and toxicity analysis of a reactive textile diazo dye- Direct Red 81 by newly isolated *Bacillus* sp. DMS2**

Shivani Amin^a^, Rajesh Prasad Rastogi^a§*^, Mukesh Ghanshyam Chaubey^b^, Kunal Jain^a^, Jyoti Divecha^c^, Chirayu Desai^d^ and Datta Madamwar^ad*^

**Supplementary Table 1:** Medium components and their high and low concentrations used in Plackett- Burman design

|  | **COMPONENTS** | **NEGATIVE (%)** | **POSITIVE** **(%)** |
| --- | --- | --- | --- |
| **X1** | Glucose | 0.1 | 1 |
| **X2** | Sucrose | 0.1 | 1 |
| **X3** | Glycine | 0.1 | 1 |
| **X4** | Glycerol | 0.1 | 1 |
| **X5** | Starch | 0.05 | 0.5 |
| **X6** | Lactose | 0.1 | 1 |
| **X7** | Yeast Extract | 0.1 | 1 |
| **X8** | Urea | 0.1 | 1 |
| **X9** | Tryptone | 0.1 | 1 |
| **X10** | Peptone | 0.1 | 1 |
| **X11** | Beef extract | 0.1 | 1 |
| **X12** | MgSO_4_ | 0.002 | 0.02 |
| **X13** | CaCl_2_ | 0.002 | 0.02 |
| **X14** | Monopotassium Phosphate | 0.01 | 0.1 |
| **X15** | Dipotassium Phosphate | 0.01 | 0.1 |
| **X16** | Ammonium Nitrate | 0.01 | 0.1 |
| **X17** | Sodium Citrate | 0.05 | 0.5 |
| **X18** | Ammonium Bicarbonate | 0.01 | 0.1 |
| **X19** | Potassium Nitrate | 0.01 | 0.1 |
| **X20** | Sodium Acetate | 0.01 | 0.1 |

**Supplementary Table 2:** Plackett- Burman design generated by fractional rotation of full factorial design where X1…………X20 are independent variable and D1…….D3 are dummy variable.

| **Run** | **X1** | **X2** | **X3** | **X4** | **X5** | **X6** | **X7** | **X8** | **X9** | **X10** | **X11** | **X12** | **X13** | **X14** | **X15** | **X16** | **X17** | **X18** | **X19** | **X20** | **D1** | **D2** | **D3** |
| --- | --- | --- | --- | --- | --- | --- | --- | --- | --- | --- | --- | --- | --- | --- | --- | --- | --- | --- | --- | --- | --- | --- | --- |
| **1** | +1 | +1 | +1 | +1 | +1 | -1 | +1 | -1 | +1 | +1 | -1 | -1 | +1 | +1 | -1 | -1 | +1 | -1 | +1 | -1 | -1 | -1 | -1 |
| **2** | -1 | +1 | +1 | +1 | +1 | +1 | -1 | +1 | -1 | +1 | +1 | -1 | -1 | +1 | +1 | -1 | -1 | +1 | -1 | +1 | -1 | -1 | -1 |
| **3** | -1 | -1 | +1 | +1 | +1 | +1 | +1 | -1 | +1 | -1 | +1 | +1 | -1 | -1 | +1 | +1 | -1 | -1 | +1 | -1 | +1 | -1 | -1 |
| **4** | -1 | -1 | -1 | +1 | +1 | +1 | +1 | +1 | -1 | +1 | -1 | +1 | +1 | -1 | -1 | +1 | +1 | -1 | -1 | +1 | -1 | +1 | -1 |
| **5** | -1 | -1 | -1 | -1 | +1 | +1 | +1 | +1 | +1 | -1 | +1 | -1 | +1 | +1 | -1 | -1 | +1 | +1 | -1 | -1 | +1 | -1 | +1 |
| **6** | +1 | -1 | -1 | -1 | -1 | +1 | +1 | +1 | +1 | +1 | -1 | +1 | -1 | +1 | +1 | -1 | -1 | +1 | +1 | -1 | -1 | +1 | -1 |
| **7** | -1 | +1 | -1 | -1 | -1 | -1 | +1 | +1 | +1 | +1 | +1 | -1 | +1 | -1 | +1 | +1 | -1 | -1 | +1 | +1 | -1 | -1 | +1 |
| **8** | +1 | -1 | +1 | -1 | -1 | -1 | -1 | +1 | +1 | +1 | +1 | +1 | -1 | +1 | -1 | +1 | +1 | -1 | -1 | +1 | +1 | -1 | -1 |
| **9** | -1 | +1 | -1 | +1 | -1 | -1 | -1 | -1 | +1 | +1 | +1 | +1 | +1 | -1 | +1 | -1 | +1 | +1 | -1 | -1 | +1 | +1 | -1 |
| **10** | -1 | -1 | +1 | -1 | +1 | -1 | -1 | -1 | -1 | +1 | +1 | +1 | +1 | +1 | -1 | +1 | -1 | +1 | +1 | -1 | -1 | +1 | +1 |
| **11** | +1 | -1 | -1 | +1 | -1 | +1 | -1 | -1 | -1 | -1 | +1 | +1 | +1 | +1 | +1 | -1 | +1 | -1 | +1 | +1 | -1 | -1 | +1 |
| **12** | +1 | +1 | -1 | -1 | +1 | -1 | +1 | -1 | -1 | -1 | -1 | +1 | +1 | +1 | +1 | +1 | -1 | +1 | -1 | +1 | +1 | -1 | -1 |
| **13** | -1 | +1 | +1 | -1 | -1 | +1 | -1 | +1 | -1 | -1 | -1 | -1 | +1 | +1 | +1 | +1 | +1 | -1 | +1 | -1 | +1 | +1 | -1 |
| **14** | -1 | -1 | +1 | +1 | -1 | -1 | +1 | -1 | +1 | -1 | -1 | -1 | -1 | +1 | +1 | +1 | +1 | +1 | -1 | +1 | -1 | +1 | +1 |
| **15** | +1 | -1 | -1 | +1 | +1 | -1 | -1 | +1 | -1 | +1 | -1 | -1 | -1 | -1 | +1 | +1 | +1 | +1 | +1 | -1 | +1 | -1 | +1 |
| **16** | +1 | +1 | -1 | -1 | +1 | +1 | -1 | -1 | +1 | -1 | +1 | -1 | -1 | -1 | -1 | +1 | +1 | +1 | +1 | +1 | -1 | +1 | -1 |
| **17** | -1 | +1 | +1 | -1 | -1 | +1 | +1 | -1 | -1 | +1 | -1 | +1 | -1 | -1 | -1 | -1 | +1 | +1 | +1 | +1 | +1 | -1 | +1 |
| **18** | +1 | -1 | +1 | +1 | -1 | -1 | +1 | +1 | -1 | -1 | +1 | -1 | +1 | -1 | -1 | -1 | -1 | +1 | +1 | +1 | +1 | +1 | -1 |
| **19** | -1 | +1 | -1 | +1 | +1 | -1 | -1 | +1 | +1 | -1 | -1 | +1 | -1 | +1 | -1 | -1 | -1 | -1 | +1 | +1 | +1 | +1 | +1 |
| **20** | +1 | -1 | +1 | -1 | +1 | +1 | -1 | -1 | +1 | +1 | -1 | -1 | +1 | -1 | +1 | -1 | -1 | -1 | -1 | +1 | +1 | +1 | +1 |
| **21** | +1 | +1 | -1 | +1 | -1 | +1 | +1 | -1 | -1 | +1 | +1 | -1 | -1 | +1 | -1 | +1 | -1 | -1 | -1 | -1 | +1 | +1 | +1 |
| **22** | +1 | +1 | +1 | -1 | +1 | -1 | +1 | +1 | -1 | -1 | +1 | +1 | -1 | -1 | +1 | -1 | +1 | -1 | -1 | -1 | -1 | +1 | +1 |
| **23** | +1 | +1 | +1 | +1 | -1 | +1 | -1 | +1 | +1 | -1 | -1 | +1 | +1 | -1 | -1 | +1 | -1 | +1 | -1 | -1 | -1 | -1 | +1 |
| **24** | -1 | -1 | -1 | -1 | -1 | -1 | -1 | -1 | -1 | -1 | -1 | -1 | -1 | -1 | -1 | -1 | -1 | -1 | -1 | -1 | -1 | -1 | -1 |

**Supplementary Table 3:** CCD codes and level values of five different variables

|  | **-2** | **-1** | **0** | **1** | **2** |
| --- | --- | --- | --- | --- | --- |
| MgSO_4_ | 0.002 | 0.0064 | 0.011 | 0.0156 | 0.02 |
| Glucose | 0.01 | 0.032 | 0.055 | 0.078 | 0.1 |
| Urea | 0.1 | 0.32 | 0.55 | 0.78 | 1 |
| pH | 4 | 5 | 7 | 9 | 11 |
| Temperature | 25 | 35 | 45 | 55 | 65 |

**Supplementary Table 4:** Experimental design of CCD with predicted and experimental values

| **Run** | **MgSO_4_ (%)** | **Glucose (%)** | **Urea (%)** | **pH** | **Tem.** | **% Decolorization Predicted** | **% Decolorization** |
| --- | --- | --- | --- | --- | --- | --- | --- |
| 1 | 0.0064 | 0.032 | 0.32 | 5 | 35 | 52.73 | 58.06 |
| 2 | 0.0156 | 0.078 | 0.32 | 5 | 35 | 87.72 | 87.16 |
| 3 | 0.0156 | 0.032 | 0.78 | 5 | 35 | 47.07 | 42.64 |
| 4 | 0.0064 | 0.078 | 0.78 | 5 | 35 | 93.29 | 97.41 |
| 5 | 0.0156 | 0.032 | 0.32 | 9 | 35 | 60.80 | 65.70 |
| 6 | 0.0064 | 0.078 | 0.32 | 9 | 35 | 86.79 | 96.03 |
| 7 | 0.0064 | 0.032 | 0.78 | 9 | 35 | 40.88 | 40.48 |
| 8 | 0.0156 | 0.078 | 0.78 | 9 | 35 | 85.69 | 90.94 |
| 9 | 0.0156 | 0.032 | 0.32 | 5 | 55 | 7.45 | 3.01 |
| 10 | 0.0064 | 0.078 | 0.32 | 5 | 55 | 13.46 | 21.06 |
| 11 | 0.0064 | 0.032 | 0.78 | 5 | 55 | 23.79 | 22.34 |
| 12 | 0.0156 | 0.078 | 0.78 | 5 | 55 | 27.04 | 29.22 |
| 13 | 0.0064 | 0.032 | 0.32 | 9 | 55 | 18.70 | 11.01 |
| 14 | 0.0156 | 0.078 | 0.32 | 9 | 55 | 26.74 | 8.11 |
| 15 | 0.0156 | 0.032 | 0.78 | 9 | 55 | 12.94 | 14.48 |
| 16 | 0.0064 | 0.078 | 0.78 | 9 | 55 | 24.11 | 22.37 |
| 17 | 0.0156 | 0.032 | 0.32 | 5 | 35 | 51.29 | 54.73 |
| 18 | 0.0064 | 0.078 | 0.32 | 5 | 35 | 84.82 | 77.20 |
| 19 | 0.0064 | 0.032 | 0.78 | 5 | 35 | 53.60 | 61.95 |
| 20 | 0.0156 | 0.078 | 0.78 | 5 | 35 | 91.11 | 97.35 |
| 21 | 0.0064 | 0.032 | 0.32 | 9 | 35 | 54.81 | 53.00 |
| 22 | 0.0156 | 0.078 | 0.32 | 9 | 35 | 97.11 | 97.75 |
| 23 | 0.0156 | 0.032 | 0.78 | 9 | 35 | 41.77 | 39.18 |
| 24 | 0.0064 | 0.078 | 0.78 | 9 | 35 | 80.45 | 82.10 |
| 25 | 0.0064 | 0.032 | 0.32 | 5 | 55 | 12.26 | 13.39 |
| 26 | 0.0156 | 0.078 | 0.32 | 5 | 55 | 18.61 | 13.00 |
| 27 | 0.0156 | 0.032 | 0.78 | 5 | 55 | 15.67 | 13.89 |
| 28 | 0.0064 | 0.078 | 0.78 | 5 | 55 | 30.92 | 32.59 |
| 29 | 0.0156 | 0.032 | 0.32 | 9 | 55 | 28.84 | 21.31 |
| 30 | 0.0064 | 0.078 | 0.32 | 9 | 55 | 28.09 | 19.78 |
| 31 | 0.0064 | 0.032 | 0.78 | 9 | 55 | 25.67 | 15.42 |
| 32 | 0.0156 | 0.078 | 0.78 | 9 | 55 | 29.15 | 25.97 |
| 33 | 0.02 | 0.055 | 0.55 | 7 | 45 | 50.64 | 54.81 |
| 34 | 0.002 | 0.055 | 0.55 | 7 | 45 | 44.12 | 54.39 |
| 35 | 0.011 | 0.1 | 0.55 | 7 | 45 | 37.49 | 51.86 |
| 36 | 0.011 | 0.01 | 0.55 | 7 | 45 | 3.66 | 7.72 |
| 37 | 0.011 | 0.055 | 1 | 7 | 45 | 0.19 | 13.77 |
| 38 | 0.011 | 0.055 | 0.1 | 7 | 45 | 8.82 | 13.67 |
| 39 | 0.011 | 0.055 | 0.55 | 11 | 45 | 10.04 | 18.80 |
| 40 | 0.011 | 0.055 | 0.55 | 4 | 45 | 1.14 | 10.11 |
| 41 | 0.011 | 0.055 | 0.55 | 7 | 65 | 2.09 | 6.93 |
| 42 | 0.011 | 0.055 | 0.55 | 7 | 25 | 98.16 | 97.02 |
| 43 | 0.011 | 0.055 | 0.55 | 7 | 45 | 10.51 | 0.47 |
| 44 | 0.011 | 0.055 | 0.55 | 7 | 45 | 0.83 | 0.47 |
| 45 | 0.011 | 0.055 | 0.55 | 7 | 45 | 4.30 | 0.47 |
| 46 | 0.011 | 0.055 | 0.55 | 7 | 45 | 2.17 | 0.47 |
| 47 | 0.011 | 0.055 | 0.55 | 7 | 45 | 1.17 | 0.47 |

**Supplementary Table S5:** Analyzing the effect of dye and metabolites on *L. minor*

|  | **% viability** | **Dry weight (g)** | | **Photosynthetic apparatus** | |
| --- | --- | --- | --- | --- | --- |
|  | **after 10 d** | **0 d** | **10 d** | **Chlorophyll-a**  **(µg/ml)** | **Chlorophyll-b**  **(µg/ml)** |
| **Control** | 99.00 ± 1.00 | 0.03 ± 0.05 | 0.06 ± 0.03 | 11.25 ± 0.39 | 3.94 ± 0.07 |
| **Product** | 98.00 ± 1.00 | 0.03 ± 0.02 | 0.08 ± 0.05 | 9.89 ± 0.18 | 3.25 ± 0.27 |
| **Dye** | 26.66 ± 3.05 | 0.03 ± 0.01 | 0.06 ± 0.01 | 7.00 ± 0.20 | 2.80 ± 0.17 |

Note: Data presented as Value ± SEM
